# Overall survival with beraprost in dogs with IRIS stage 2 chronic kidney disease

**DOI:** 10.3389/fvets.2026.1665061

**Published:** 2026-04-24

**Authors:** Masayuki Mayumi, Takumi Matsuura, Tadashi Sano

**Affiliations:** 1 Fujisawa Yui Animal Clinic, Fujisawa, Kanagawa, Japan; 2 Obihiro University of Agriculture and Veterinary Medicine, Obihiro, Hokkaido, Japan

**Keywords:** beraprost, canine, chronic kidney disease, overall survival, prostacyclin analog

## Abstract

Chronic kidney disease (CKD) in dogs is a progressive condition with a poor prognosis and a lack of effective pharmacological interventions to extend survival. Beraprost, an oral prostacyclin analog approved for feline CKD, was off-label prescribed and evaluated for its effect on survival in dogs with naturally occurring IRIS stage 2 CKD. This prospective clinical study included a treatment group (*n* = 16) receiving oral beraprost (12.5 μg/kg twice daily), and a well-matched historical control group (*n* = 17) from the same institution. The primary outcome, overall survival, was analyzed using the Kaplan-Meier method and a multivariable Cox proportional hazards model. Baseline characteristics were largely comparable between the groups. Significantly longer survival was observed in the beraprost-treated group, with a median survival time of 1,101 days (a 5.6-fold increase), compared to 198 days in the control group (*P* = 0.001). Furthermore, beraprost therapy was associated with a significant delay in the time to secondary composite endpoints, including disease progression (to IRIS stage 3/4) or death (*P* = 0.001) and 10% body weight loss or death (*P* = 0.028), underscoring the robustness of the survival data. After adjusting for age and urine protein-to-creatinine ratio, beraprost treatment remained the sole independent predictor of improved survival (hazard ratio, 0.15; 95% confidence interval 0.03–0.79; *P* = 0.025). Although limited by its non-randomized design and small sample size, this study provides the first clinical evidence that oral beraprost is associated with a substantial benefit on overall survival in dogs with IRIS stage 2 CKD, supporting the need for large-scale randomized controlled trials.

## 1 Introduction

Chronic kidney disease (CKD) is a common disease in older dogs and a leading cause of morbidity and mortality (1–3). It is defined by the presence of functional and/or structural kidney abnormalities that persist for at least 3 months (4). CKD's pathophysiology is multifactorial, involving a progressive loss of nephrons, leading to compensatory glomerular hyperfiltration, systemic and intraglomerular hypertension, and subsequent tubulointerstitial inflammation and fibrosis, ultimately culminating in end-stage renal disease (5). Management of canine CKD has primarily focused on supportive and symptomatic treatments. These include renal therapeutic diets to control uremia and hyperphosphatemia, phosphate binders, antihypertensive drugs like angiotensin-converting enzyme inhibitors (ACEIs) to manage systemic hypertension and reduce proteinuria, and fluid therapy to correct dehydration (4, 6–8). While nutritional management with renal therapeutic diets has been shown to improve survival (9), specific pharmacological interventions that significantly prolong survival in dogs with naturally occurring CKD are limited.

Beraprost, a stable and orally active prostacyclin (PGI2) analog, is a promising treatment for kidney disease due to its various pharmacological effects. These effects include vasodilation, inhibition of platelet aggregation, anti-inflammatory actions, and cell protection (10, 11). In rodent models of CKD, such as 5/6 nephrectomy and anti-glomerular basement membrane (anti-GBM) glomerulonephritis, beraprost shows protective effects on the kidneys, leading to improved survival rates. The treatment slows the decline in kidney function and preserves the renal microvascular network, partly by inhibiting mitochondria-dependent apoptosis in renal cells (12, 13). Additionally, in models of diabetic nephropathy, beraprost has been demonstrated to reduce proteinuria, suppress glomerular macrophage influx, and reduce sclerotic changes (14). Furthermore, recent research has highlighted its endothelial protective properties, demonstrating that beraprost improves microcirculation and barrier function by ameliorating oxidative stress imbalances in models of renal failure (15).

Beraprost, a stable prostacyclin analog, has been approved in Japan for managing feline CKD, providing a novel therapeutic option for its management. This approval was granted primarily based on a pivotal multicenter, prospective, randomized, double-blind, placebo-controlled trial. The study demonstrated that in cats with IRIS CKD stages 2 and 3, oral beraprost was well-tolerated and effectively slowed the progression of renal dysfunction, as assessed by serum creatinine concentration, over a 180-day period (16). Further research has provided additional evidence supporting its clinical utility. A retrospective cohort study in a real-world setting reported that beraprost therapy was associated with significantly prolonged overall survival in cats with advanced CKD (17). Moreover, its mechanism of action has been further elucidated, revealing that beraprost reduces plasma aldosterone concentration through a nitric oxide synthase (NOS)-dependent pathway, without significantly altering systemic hemodynamics in healthy cats (18).

It is crucial to recognize that the etiology and pathogenesis of CKD differ among animal species. For instance, primary glomerulopathies are more prevalent in dogs, while tubulointerstitial disease is the predominant form in cats (2, 19). Given these pathophysiological variations, the therapeutic effects of a drug cannot be extrapolated from one species to another. Thus, the kidney-protective effects of beraprost found in rodents and cats are not guaranteed in dogs, which reinforces the need for specific research in the canine species.

Previous preclinical studies have established the safety and pharmacokinetic profiles of beraprost in dogs (20, 21). For instance, a 12-month oral chronic toxicity study in healthy dogs determined the no-observed-adverse-effect level (NOAEL) to be 25 μg/kg/day (20). More recently, a pharmacodynamic study in canine models of pulmonary hypertension found that 25 μg/kg of beraprost administered twice daily (50 μg/kg/day) influenced systemic cardiovascular hemodynamics (22). Furthermore, a subsequent clinical study in 16 dogs with naturally occurring pulmonary hypertension demonstrated that continuous administration of beraprost (range: 13.2–22.0 μg/kg) significantly decreased pulmonary and systemic vascular impedance and increased left and right ventricular myocardial strain, with no observed side effects (23).

This prospective clinical study aimed to investigate the effect of off-label beraprost use on the prognosis of dogs with naturally occurring IRIS stage 2 CKD. Based on the hypothesis that beraprost would improve survival, the authors compared outcomes from the treatment group with those of a well-matched historical control group from the same institution.

## 2 Materials and methods

### 2.1 Study design and ethical considerations

This was a prospective clinical study with a historical control group conducted at the Fujisawa Yui Animal Clinic. This study was conducted in accordance with the principles of the Declaration of Helsinki. The protocol was reviewed and approved by the Institutional Animal Care and Use Committee (IACUC) of the Fujisawa Yui Animal Clinic, which serves as both the Ethics Committee and the Animal Welfare Body for this institution (Approval No. 2020001). All procedures followed institutional and national guidelines for the care and use of animals. Before enrolling dogs in the prospective treatment group, written informed consent was obtained from all owners.

### 2.2 Animals

Client-owned dogs with a diagnosis of naturally occurring CKD were included in the study. The diagnosis of CKD was based on a serum creatinine level persistently above the reference range, and a urine specific gravity (USG) of < 1.030. This was supported by the dog's clinical history and/or findings from diagnostic imaging. For the patient to be eligible, these laboratory abnormalities had to be present on at least two separate occasions at least 4 weeks apart.

For this study, all dogs had to be classified as IRIS CKD stage 2 (serum creatinine 1.4–2.8 mg/dl) at the time of inclusion, based on the IRIS CKD guidelines (4) available at the time of diagnosis. Although the IRIS CKD guidelines were updated during the overall study period, staging was consistently performed using the most current version available. Dogs with other health problems that could significantly shorten their life expectancy unrelated to CKD (e.g., advanced-stage cancer) or those with a history of acute kidney injury were excluded.

A total of 33 dogs that met these criteria were included in the analysis and assigned to one of two groups:

1. The historical control group (*n* = 17) consisted of dogs identified through a retrospective medical record search. The authors reviewed the records of all dogs diagnosed with IRIS stage 2 CKD managed at the clinic between April 27, 2018, and January 4, 2020. All dogs meeting the same inclusion and exclusion criteria during this period were deemed eligible, resulting in the inclusion of 17 dogs.
2. The prospective therapy group (*n* = 16) included dogs meeting these criteria who were enrolled on or after January 5, 2020.

The follow-up period for all dogs concluded on May 1, 2025.

### 2.3 Data collection and outcomes

In the Fujisawa Yui Animal Clinic, key clinical data, including laboratory results, treatment histories, and survival outcomes, were maintained in a structured electronic database, which enabled robust and reliable data extraction for this study. The start date for the analysis was the date of the first beraprost prescription for the therapy group, or the date of IRIS CKD staging for the historical control group. Baseline data, including age, body weight, sex, breed, clinical pathology results (serum creatinine, urea, phosphate, calcium, potassium, albumin, and hematocrit), urinalysis (USG and urine protein-to-creatinine ratio [UPCR]), and systolic blood pressure, were collected from medical records on or before the start date. Any coexisting disorders and concurrent treatments were also recorded. During the follow-up period, to screen for potential adverse effects, owners were instructed to carefully monitor their dogs at home for any abnormal clinical signs and to report them to the clinic immediately. Additionally, routine physical examinations and laboratory tests were conducted during scheduled follow-up visits to actively assess patient safety.

The primary outcome was overall survival, measured from the start date to the date of death from any cause. For this primary analysis, dogs that were lost to follow-up or were still alive at the end of the study period (May 1, 2025) were censored. Secondary outcomes were evaluated using two composite endpoints, defined as the time from the start date to the first occurrence of the event. The first composite endpoint (progression or death) was defined as either persistent progression to IRIS stage 3 (serum creatinine persistently between 2.9 and 5.0 mg/dl) or IRIS stage 4 (serum creatinine persistently >5.0 mg/dl), based on the IRIS CKD guidelines [e.g., IRIS (4)], or death from any cause, whichever occurred first. The second composite endpoint (10% body weight loss or death) was defined as either a 10% reduction in body weight from baseline (as a surrogate marker for cachexia and quality of life) or death from any cause, whichever occurred first. For these composite endpoint analyses, dogs that were lost to follow-up, or were still alive without having reached the respective endpoint at the end of the study period, were censored.

### 2.4 Treatment

Although beraprost is approved in Japan for the treatment of feline CKD, its use in dogs for this study was off-label. The dosage was selected based on prior safety and pharmacodynamic data to minimize potential hemodynamic side effects (20, 22, 23). Therefore, based on these findings, a target dose of 12.5 μg/kg administered twice daily after feeding was selected for the present study in dogs with CKD, and the post-feeding administration protocol was consistent with that previously established for cats. Beraprost was administered continuously (lifelong) unless the owners elected to discontinue treatment or the dog was euthanized or died. The actual administered doses are presented in Table 1. Dogs in the historical control group did not receive beraprost. Standard-of-care therapies for CKD, such as renal therapeutic diets, subcutaneous fluid therapy, phosphate binders, and angiotensin-converting enzyme inhibitors (ACEIs), were provided to dogs in both groups as clinically indicated by the attending veterinarian.

**Table 1:** Baseline characteristics of dogs with chronic kidney disease (CKD) in the Beraprost therapy group (*n* = 16) and the No Beraprost therapy (historical control) group (*n* = 17).

	Summary statistics	Total (*n* = 33)	Beraprost therapy (*n* = 16)	No beraprost therapy (*n* = 17)	*P*-value
Parameters					
Age (years)	Median (IQR)	14.8 (13.7, 15.4)	14.0 (13.4, 14.7)	15.1 (14.8, 16.0)	0.044^*^
15.6-7.5,0498ptWeight (kg)	Median (IQR)	3.8 (2.8, 6.3)	4.1 (2.9, 6.6)	3.8 (2.6, 6.1)	0.640
Sex					
Female	Number (%)	25 (75.8%)	12 (75.0%)	13 (76.5%)	1.000
15.6-7.5,0498ptMale	Number (%)	8 (24.2%)	4 (25.0%)	4 (23.5%)	
Reproductive status					
Intact	Number (%)	5 (15.2%)	2 (12.5%)	3 (17.6%)	1.000
15.6-7.5,0498ptNeutered	Number (%)	28 (84.8%)	14 (87.5%)	14 (82.4%)	
Breed					
Toy poodle	Number (%)	9 (27.2%)	5 (31.3%)	4 (23.5%)	0.708
Chihuahua	Number (%)	4 (12.1%)	2 (12.5%)	2 (11.8%)	
Dachshund	Number (%)	4 (12.1%)	2 (12.5%)	2 (11.8%)	
Mixed breed	Number (%)	4 (12.1%)	2 (12.5%)	2 (11.8%)	
Shetland Sheepdog	Number (%)	2 (6.1%)	1 (6.3%)	1 (5.9%)	
15.6-7.5,0498ptOther	Number (%)	10 (30.3%)	4 (25.0%)	6 (35.3%)	
Biochemistry					
Creatinine (mg/dl)	Median (IQR)	1.6 (1.5, 2.1)	1.7 (1.5, 2.1)	1.6 (1.6, 1.8)	0.859
Urea (mg/dl)	Median (IQR)	49.2 (43.4, 72.2)	47.5 (42.5, 69.3)	51.4 (44.9, 72.2)	0.978
Phosphate (mg/dl)	Median (IQR)	4.8 (3.9, 6.2)	4.8 (4.1, 5.7)	5.7 (3.4, 7.6)	0.932
Calcium (mg/dl)	Median (IQR)	11.6 (11.0, 11.9)	11.7 (11.0, 12.2)	11.3 (11.2, 11.5)	0.560
Potassium (mmol/L)	Median (IQR)	4.4 (3.8, 4.8)	4.6 (3.8, 4.8)	4.4 (3.9, 4.9)	0.853
15.6-7.5,0498ptAlbumin (g/dl)	Median (IQR)	3.2 (2.9, 3.6)	3.3 (3.2, 3.4)	3.1 (2.7, 3.6)	0.507
Hematology					
15.6-7.5,0498ptHematocrit (%)	Median (IQR)	48.1 (43.5, 52.4)	49.9 (47.6, 52.5)	46.1 (37.8, 50.0)	0.155
Urinalysis					
Urine specific gravity	Median (IQR)	1.016 (1.014, 1.024)	1.014 (1.014, 1.022)	1.020 (1.016, 1.024)	0.252
15.6-7.5,0498ptUrine protein-to-creatinine ratio	Median (IQR)	0.35 (0.01, 0.35)	0.35 (0.01, 0.45)	0.02 (0.02, 0.35)	0.162
Blood pressure measurement					
Systolic blood pressure (mmHg)	Median (IQR)	153 (148, 173)	151 (148, 157)	173 (159, 183)	0.606
15.6-7.5,0498ptMean blood pressure (mmHg)	Median (IQR)	107 (103, 125)	107 (100, 123)	128 (117, 138)	0.245
Treatment					
Beraprost	Number (%)	16 (48.5%)	16 (100.0%)	0 (0.0%)	< 0.001^**^
Dose of beraprost (μg/kg twice daily)	Mean (SD, range)	13.1 (1.9, 9.8-15.9)	13.1 (1.9, 9.8-15.9)	0 (0, 0-0)	< 0.001^**^
Subcutaneous fluid therapy	Number (%)	24 (72.7%)	13 (81.3%)	11 (64.7%)	0.438
Phosphate binder	Number (%)	6 (18.2%)	4 (25.0%)	2 (11.8%)	0.398
15.6-7.5,0498ptACEI (Benazepril)	Number (%)	3 (9.1%)	1 (6.3%)	2 (11.8%)	1.000
Coexisting disorders (multiple counts per patient)					
Any	Number (%)	28 (84.8%)	13 (81.3%)	15 (88.2%)	0.656
Cholangiohepatitis	Number (%)	8 (24.2%)	4 (25.0%)	4 (23.5%)	1.000
Myxomatous mitral valve disease	Number (%)	4 (12.1%)	2 (12.5%)	2 (11.8%)	1.000
Tracheal collapse	Number (%)	4 (12.1%)	3 (18.8%)	1 (5.9%)	0.335
Epilepsy	Number (%)	3 (9.1%)	0 (0.0%)	3 (17.6%)	0.227
Inflammatory bowl disease	Number (%)	2 (6.1%)	1 (6.3%)	1 (5.9%)	1.000
Chronic pancreatitis	Number (%)	1 (3.0%)	1 (6.3%)	0 (0.0%)	0.485
Congestive heart failure	Number (%)	1 (3.0%)	0 (0.0%)	1 (5.9%)	1.000
Cushing's syndrome	Number (%)	1 (3.0%)	0 (0.0%)	1 (5.9%)	1.000
Cutaneous lymphoma	Number (%)	1 (3.0%)	1 (6.3%)	0 (0.0%)	0.485
Diabetes mellitus	Number (%)	1 (3.0%)	0 (0.0%)	1 (5.9%)	1.000
Hypertension	Number (%)	1 (3.0%)	1 (6.3%)	0 (0.0%)	0.485
Hypothyroidism	Number (%)	1 (3.0%)	0 (0.0%)	1 (5.9%)	1.000

Continuous variables are presented as median (IQR), with the exception of the Beraprost dose, which is shown as mean (SD, range). Categorical variables are presented as number (%).

P-values for the comparison between the two groups were calculated using Welch's t-test (for creatinine, urea, and urine protein-to-creatinine ratio), the Mann–Whitney U-test (for other continuous variables), and Fisher's exact test (for categorical variables).

ACEI, angiotensin-converting enzyme inhibitor; SD, standard deviation.

^*^P < 0.05; ^**^P < 0.01.

### 2.5 Statistical analysis

Baseline characteristics were compared between the two groups. Quantitative variables were presented as median and respective interquartile range (IQR), and compared using the Mann–Whitney *U*-test, or Welch's *t*-test for variables like creatinine, urea, and UPCR, as specified. Qualitative variables were presented as number (%) and compared using the Chi-square test or Fisher's exact test, as appropriate.

Overall survival was assessed with the Kaplan–Meier method, and survival curves were compared between the groups using the log-rank test. To identify prognostic factors for all-cause mortality while adjusting for other variables, a multivariable Cox proportional hazards model was constructed using a forward stepwise selection procedure. Covariates (beraprost treatment, age, and UPCR) with a *P*-value < 0.20 in the univariate analysis were retained for the final model. All covariates were included as continuous variables. The proportional-hazards assumption was assessed by inspecting log-log plots of adjusted survival curves. Hazard ratios (HRs) and their 95% confidence intervals (CI) were calculated, with *P*-values determined by the Wald test.

All statistical analyses were performed using BellCurve for Excel (Social Survey Research Information Co., Ltd., Tokyo, Japan). A two-sided *P*-value of < 0.05 was considered statistically significant.

## 3 Results

A total of 33 client-owned dogs with CKD were included in this study. The dogs were divided for analysis into a beraprost therapy group (16 dogs) and a no beraprost therapy (historical control) group (17 dogs).

Table 1 shows the baseline characteristics for both groups. The two groups were well-matched for most variables, but there was a significant difference in age. The historical control group was older (median, 15.1 years [IQR: 14.8–16.0]) compared to the beraprost therapy group (median, 14.0 years [IQR: 13.4–14.7]; *P* = 0.044). Of note, there were no significant differences between the groups in key prognostic markers for CKD, like serum creatinine (*P* = 0.859), urine protein-to-creatinine ratio (UPCR; *P* = 0.162), or systolic blood pressure (*P* = 0.606). The number of dogs with coexisting disorders and the use of concurrent treatments, such as subcutaneous fluids and angiotensin-converting enzyme inhibitors (ACEIs), were also comparable.

**Figure 1:**
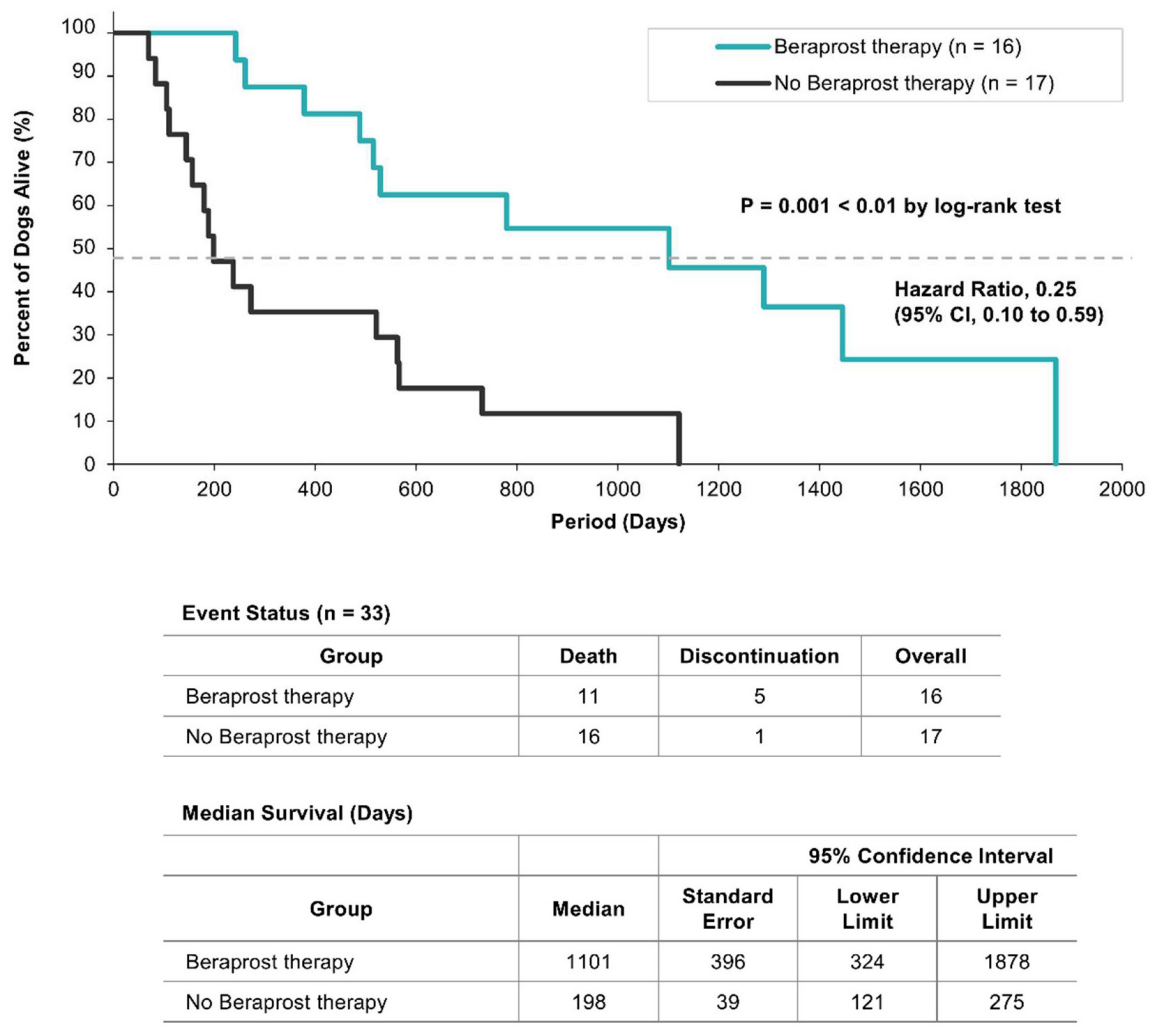
Kaplan–Meier survival curves for dogs with chronic kidney disease (CKD). The survival probability of dogs receiving beraprost therapy is compared to that of the no beraprost therapy group (historical controls). The statistical comparison between the groups was performed using a log-rank test. CI, confidence interval.

Kaplan-Meier analysis showed that overall survival was significantly longer with beraprost therapy compared to no therapy (*P* = 0.001; Figure 1). The median overall survival was 1,101 days (95% CI, 324–1,878 days) for the beraprost group, and 198 days (95% CI, 121–275 days) for the no-therapy group. This corresponds to an unadjusted HR of 0.25 (95% CI, 0.10–0.59), representing a 75% reduction in the risk of all-cause mortality for dogs treated with beraprost.

Two secondary endpoints were evaluated: time to disease progression (to IRIS stage 3 or 4) or death from any cause, and time to 10% body weight loss from baseline or death from any cause. Beraprost therapy was associated with a significant extension of the time to the occurrence of both the progression and the body weight loss endpoints (*P* = 0.001 and *P* = 0.028, respectively). The median event-free survival times for both groups are detailed in Table 2.

**Table 2:** Median event-free survival for secondary endpoints.

			95% confidence interval		
	**Median**	**Standard error**	**Lower limit**	**Upper limit**	***P*** **-value**
Group					
Median event-free survival of IRIS stage progression (Days)					
Beraprost therapy	1,101	396	324	1,878	0.001^**^
No Beraprost therapy	179	42	97	261	
Median event-free survival of body weight loss (Days)					
Beraprost therapy	403	79	247	559	0.028^*^
No Beraprost therapy	142	26	90	194	

^*^P < 0.05; ^**^P < 0.01.

To exclude confounding factors that could influence the treatment effect on survival, a multivariable Cox proportional hazards model was constructed using baseline variables such as age and UPCR, with beraprost as a covariate (Table 3). The analysis identified one factor that correlated significantly with improved survival; the HR for beraprost was 0.15 (95% CI, 0.03–0.79; *P* = 0.025). This indicates that after adjusting for other variables, beraprost was the only treatment associated with a significantly better prognosis. In contrast, neither age (HR, 1.45; *P* = 0.183) nor UPCR (HR, 13.90; *P* = 0.083) showed a statistically significant correlation with mortality in this model.

**Table 3:** Multivariable Cox proportional hazards analysis for all-cause mortality in dogs with chronic kidney disease (CKD).

				95% confidence interval		
				**Lower limit**	**Upper limit**	
**Covariate**	**Coefficient**	**Standard error**	**Hazard ratio for death**			*P*-value
Beraprost	−1.91	0.85	0.15	0.03	0.79	0.025^*^
Age (years)	0.37	0.28	1.45	0.84	2.51	0.183
Urine protein-to-creatinine ratio	2.63	1.52	13.90	0.71	272.74	0.083

The model includes treatment with beraprost, age, and urine protein-to-creatinine ratio as covariates.

HRs are presented with 95% confidence intervals (CI).

^*^P < 0.05 by Wald test.

The cause of death was determined for all 27 dogs that died during the study (11 in the beraprost group, 16 in the control group). Progressive CKD was the primary cause of death in the majority of cases (*n* = 7 in the beraprost group and *n* = 11 in the control group). Other non-renal causes were also recorded, which included respiratory disease (*n* = 2), infectious disease (*n* = 1), and neurological disease (*n* = 1) in the beraprost group, and autoimmune disease (*n* = 1), cardiac disease (*n* = 1), gastrointestinal disease (*n* = 1), infectious disease (*n* = 1), and neurological disease (*n* = 1) in the control group. No dogs in either group were euthanized. Furthermore, no suspected adverse effects or deaths in the therapy group were considered directly related to beraprost administration.

## 4 Discussion

The present prospective clinical study with a historical control group provides the first clinical evidence for a survival benefit of beraprost, an oral prostacyclin analog, in dogs with naturally occurring IRIS stage 2 CKD. Non-randomized studies, such as this one using a historical control group, have limitations compared to randomized controlled trials (RCTs). However, they can provide valuable information on treatment effects in real-world clinical practice, complementing evidence from RCTs. The main finding of this study is that the oral administration of beraprost was associated with a statistically significant improvement in overall survival: the median survival time in the beraprost group was 1,101 days, representing a 5.6-fold increase compared to 198 days in the control group. This corresponded to a 75% reduction in the risk of all-cause mortality (HR, 0.25; 95%CI, 0.10–0.59), suggesting that beraprost may be an effective therapy for the management of canine CKD.

To confirm the external validity of the present study population, it is important to compare its demographic characteristics with those of previous reports. Several characteristics of this cohort were consistent with previous studies. The predominance of small breeds in this population is consistent with the study by Perini-Perera et al. (24), where small-sized dogs comprised 66.7% of their CKD cohort. Similarly, the median serum creatinine concentration was 1.6 mg/dl, a value similar to the median of 1.7 mg/dl reported for dogs with IRIS stage 2 CKD (25). In addition, the high prevalence of comorbidities, with 84.8% of dogs having at least one other disorder, is typical for older veterinary patients with CKD.

However, this cohort also showed some differences from previous reports. The median age (14.0–15.1 years) was higher than the 10-year median reported by Perini-Perera et al. (24). Although it is known that CKD is a disease of older dogs (26), the advanced age of this study population suggests a higher risk of death, as advanced age is a significant risk factor for mortality (3). Additionally, the prevalence of several specific comorbidities in this cohort was lower than that reported in a large-scale study by O'Neill et al. (3). For instance, the prevalence of cardiac disorders (12.1% with myxomatous mitral valve disease and 3.0% with congestive heart failure) was lower than the 29.8% reported in their study. We also found a lower prevalence of hypertension [3.0% vs. 6.1% in (3)] and chronic pancreatitis [3.0% vs. 4.8% in (3)]. These differences might be due to variations in cohort characteristics or diagnostic criteria between studies. Still, the high rate of comorbidity highlights the challenge of managing canine CKD.

To evaluate the therapeutic effect of beraprost, a historical control group was used. The median survival time in this no-beraprost group was 198 days (approx. 6.6 months). This outcome is consistent with the survival times seen in dogs with IRIS stage 2 CKD, for which median survival times have been reported to range from 200 to 400 days (3, 26, 27). The survival time of the control group falls within the lower end of this reported range, which provides a baseline for this comparison and highlights the positive effect of beraprost treatment.

Conventional management strategies for canine CKD include therapeutic renal diets, which have been robustly shown to improve survival time and quality of life. Furthermore, pharmacological interventions such as angiotensin-converting enzyme (ACE) inhibitors and angiotensin receptor blockers (ARBs) are strongly recommended by current IRIS guidelines, primarily for the management of proteinuria and systemic hypertension. However, evidence for direct overall survival improvement solely from these pharmacological interventions in non-proteinuric advanced CKD remains less definitive. For example, Finco et al. (28) and Grauer et al. (6) demonstrated benefits of ACE inhibitors in glomerulonephritis models, but without conclusive evidence for increased overall survival. Similarly, antioxidant supplements, RAAS modulators, and herbal renal protectants frequently used in practice remain under-evaluated in peer-reviewed studies. In this context, the survival extension with beraprost observed in the present study population (median 1,101 vs. 198 days) confers a clinically meaningful advantage, especially given the advanced age and high comorbidity burden among the dogs enrolled. This finding is consistent with recent reports in cats with advanced CKD, where beraprost therapy was also associated with significantly prolonged survival (17), suggesting a potential disease-modifying effect of beraprost across species.

Focusing solely on survival as an endpoint in CKD studies can be a limitation, as it provides no information on disease progression itself. Dogs with CKD, particularly geriatric patients, often suffer from multiple comorbidities and may die from other causes before a significant decline in renal markers is observed (3, 24). Therefore, to provide a more comprehensive assessment, this study included a secondary analysis using a composite endpoint of CKD progression (to IRIS stage 3 or 4) or all-cause mortality. The finding that beraprost therapy significantly delayed this composite event provides crucial secondary evidence. It suggests that the substantial survival benefit observed is, at least in part, mediated by beraprost's effect on slowing the progression of renal dysfunction, thereby supporting the therapeutic hypothesis.

In addition to disease progression, this study utilized a 10% body weight loss as a surrogate marker for cachexia, a key contributor to poor quality of life (QOL) in dogs with CKD. Unintentional weight loss is strongly associated with negative outcomes in canine CKD (26). The finding that beraprost therapy significantly delayed the composite endpoint of 10% body weight loss or death is therefore clinically relevant. It suggests that the survival benefit observed with beraprost may be associated not only with slowed progression of renal dysfunction, but also with the maintenance of body condition and, by extension, a better quality of life.

A limitation of this study was the significant difference in baseline age between the groups. The group not receiving beraprost therapy was older than the beraprost-treated group. Because advanced age is a known negative prognostic factor (3), this difference at baseline could have biased the results toward the treatment group. However, in the multivariable Cox proportional hazards model, beraprost treatment was the only independent factor significantly associated with better survival, while age was not a significant predictor. This suggests it is unlikely that the age difference confounded the main finding of this study.

Notably, beraprost was well-tolerated, and no suspected adverse drug reactions were seen. The dose of 12.5 μg/kg twice daily was chosen based on prior studies to reduce potential side effects (20, 22). In a 12-month oral chronic toxicity study in healthy dogs, a toxic dose of 250 μg/kg/day was reported to cause adverse clinical signs, including diarrhea, bloody stools, and mild sedation (20). In the present study, which administered 12.5 μg/kg twice daily—a dose corresponding to the no-observed-adverse-effect level—to dogs with CKD, none of these adverse reactions were observed. This suggests that the safety margin is maintained in patients with CKD. Furthermore, the ease of oral administration (12.5 μg/kg BID after meals) and absence of observed adverse effects support its integration into conventional treatment regimens. In practice, polypharmacy, patient compliance, and owner adherence are key considerations in managing elderly CKD patients. Beraprost's tolerability and compatibility with standard therapies—including ACEIs and dietary interventions—make it a practical addition to multimodal protocols in general practice settings. Nevertheless, further pharmacokinetic and pharmacodynamic studies in dogs with CKD would be desirable to confirm the optimal dosing regimen.

This study has several other limitations. A primary limitation of the present study is its non-randomized design utilizing a historical control group. This methodology is inherently susceptible to selection bias and unmeasured confounding variables that could influence outcomes. Furthermore, because the control data were collected retrospectively, potential temporal variations in the general standard of care, diagnostic sensitivities, and owner compliance between the historical and treatment cohorts cannot be completely ruled out. First, the non-contemporaneous nature of the control group is a key limitation. Although the control period (2018–2020) immediately preceded the treatment period (2020–), time-dependent factors such as improvements in general veterinary care or increased owner awareness (the “period effect”) could theoretically favor the prospective group. However, it is worth noting that the standard clinical protocols for CKD management at the institution where the study was conducted—including the criteria for prescribing diets and antihypertensive agents—remained consistent across both periods, which may limit the magnitude of this bias. Second, the small sample size (*n* = 33) limits the study's statistical power. This may explain why the urine protein-to-creatinine ratio (UPCR), a well-established prognostic factor, did not reach statistical significance in this multivariable model. This finding may represent a type II statistical error attributable to the limited statistical power of the study. Interestingly, (24) also found no significant association between survival and proteinuria or hypertension in their cohort, possibly due to similar reasons related to sample size. Furthermore, an *a priori* statistical power analysis was not performed because the sample size was dictated by the availability of eligible clinical cases during the study period. Therefore, the present research should be considered an exploratory study. Third, serum symmetric dimethylarginine (SDMA) was not measured. Although SDMA allows for more sensitive detection of early kidney dysfunction, the disease staging relied on serum creatinine and USG, reflecting the standard of care in this clinical setting during the study period. Fourth, although baseline characteristics including breed distribution and sex were comparable between the groups (Table 1), the small sample size precluded a stratified analysis to definitively rule out confounding effects related to these variables. Finally, because the study was not blinded, there is a potential for a caregiver placebo effect. Although the standard definition of CKD requires the persistence of functional abnormalities for at least 3 months, a confirmation period of at least 4 weeks was employed as the inclusion criterion. In a clinical setting, delaying therapeutic intervention for 3 months solely to satisfy the strict definition of chronicity raises ethical concerns regarding patient welfare. Therefore, an early intervention was prioritized for dogs consistently presenting with Stage 2 markers.

## 5 Conclusion

This study provides the first clinical evidence that oral beraprost is associated with improved overall survival in dogs with IRIS stage 2 CKD. The robustness of this primary finding is supported by associated benefits on key secondary endpoints, including disease progression and body condition, suggesting a clinically relevant effect that warrants further investigation. Given its ease of use, safety profile, and potential synergy with standard therapies, beraprost could represent a valuable addition to the therapeutic landscape for canine CKD. The authors advocate for the initiation of multicenter, prospective randomized controlled trials to validate these findings and explore its role in integrated care strategies.

## Data Availability

The original contributions presented in the study are included in the article/supplementary material, further inquiries can be directed to the corresponding author.
